# Next-generation glycogen storage diseases

**DOI:** 10.1007/s10545-018-00250-0

**Published:** 2018-11-23

**Authors:** Terry G. J. Derks, Maaike H. Oosterveer, Carolina F. De Souza

**Affiliations:** 1 Section of Metabolic Diseases, Beatrix Children’s Hospital, University Medical Center of Groningen, University of Groningen, Hanzeplein 1, 9700 RB Groningen, The Netherlands; 2 Department of Pediatrics, Center for Liver Digestive and Metabolic Diseases, University Medical Center Groningen, University of Groningen, Groningen, The Netherlands; 3 Medical Genetics Service, Hospital de Clínicas de Porto Alegre, Porto Alegre, Brazil

The glycogen storage diseases (GSD) are amongst the earliest recognized inborn errors of metabolism. After the Fulda Workshop on Glycogen Storage Disease (GSD) type I in 1990, international GSD conferences have been held in Fulda (2000), Milan (2010), Lyon (2012), and Heidelberg (2013). Since the start of the internet era, parent-led patient organizations have pushed for international guidelines for treatment and follow-up (Phillips 2002). Subsequent international collaborative cohort studies have defined the phenotypes of GSDs and facilitated the development of internationally agreed clinical guidelines. This special edition of the *Journal of Inherited Metabolic Disease* discusses highlights of the International GSD conference held at the University Medical Center Groningen, the Netherlands, between 15 and 17 June 2017 (IGSD2017). Fifty-three oral presentations, 52 posters, 11 networking sessions, and 2 “Meet the Expert” sessions attended by 406 participants from 36 countries reflected the recent progress in individualized and novel therapies, monitoring, and empowerment for patients with muscle- and/or liver-specific GSD types.

To foster the involvement of all stakeholders, including parents, in setting the future research agenda, the International Liver Glycogen Storage Disease Priority Setting Partnership (IGSD-PSP) (http://igsdpsp.com) was initiated during the IGSD2017 conference, in collaboration with the James Lind Alliance (http://www.jla.nihr.ac.uk/). This partnership aims to identify and prioritize research addressing current uncertainties about the effects of care and management. The IGSD-PSP is the first international, multi-lingual PSP for a group of ultra-rare disorders. The steering group expects to present the results at the end of 2019.

A highlight of IGSD2017 was the plenary session on gene therapy, including longitudinal results after gene therapy in canine GSD Ia models. Ideally, GSD Ia patients would be no longer dependent on intensive and unsafe (nocturnal) dietary management after a single dose of gene therapy. To date, longitudinal, in vivo functional studies of glucose metabolism in animal models after gene therapy are lacking. Case histories of “mild” GSD Ia patients have demonstrated that episodes of hypoglycemia can remain undiagnosed until patients present with liver adenomas, indirectly emphasizing the importance of determining that exogenously administered genes are stably expressed. Longitudinal monitoring of GSD Ia patients after gene therapy may include a combination of (1) assessment of microsomal glucose-6-phosphatase activities ex vivo (necessitating invasive liver biopsies), (2) execution of (invasive, clinical) fasting challenges in vivo, (3) advanced continuous glucose monitoring in the home situation, and (4) application of stable isotope methods to longitudinally quantify endogenous glucose production rates in vivo (van Dijk et al. 2013).

We are delighted to present this special collection of papers about GSD. Ultra-rare inborn errors of metabolism do not stop at borders between countries or continents and global collaborations are essential. Clinical expertise, secure eHealth applications, active patient participation, and transparent governance structures including all important stakeholders will be fundamental to improving cross border transfer of knowledge, care, and cure between professionals and patients, in all possible directions.
